# Barriers experienced by and educational needs of clinicians who provide care for transgender, nonbinary, and gender-diverse young adults in the Mid-Atlantic and Southern United States

**DOI:** 10.1371/journal.pone.0326420

**Published:** 2025-06-23

**Authors:** Jessica Kremen, Meg Quint, Regina Tham, Kaiden Kane, Elizabeth R. Boskey, Cassandra Morrow, Sari L. Reisner, Rena Xu

**Affiliations:** 1 Department of Endocrinology, Boston Children’s Hospital, Boston, Massachusetts, United States of America; 2 Department of Epidemiology, University of Michigan School of Public Health, Ann Arbor, Michigan, United States of America; 3 Stanford Medical School, Stanford, California, United States of America; 4 Department of Urology, Boston Children’s Hospital, Boston, Massachusetts, United States of America; 5 Division of Gynecology, Boston Children’s Hospital, Boston, Massachusetts, United States of America; 6 Department of Surgery, Harvard Medical School, Boston, Massachusetts, United States of America; 7 Department of Medicine, Harvard Medical School, Boston, Massachusetts, United States of America; 8 Department of Epidemiology, Harvard T.H. Chan School of Public Health, Boston, Massachusetts, United States of America; 9 Department of Social and Behavioral Sciences, Harvard T.H. Chan School of Public Health, Boston, Massachusetts, United States of America; RAND Corporation, UNITED STATES OF AMERICA

## Abstract

**Purpose:**

The purpose of this study is to identify and explore the educational needs of and broader barriers experienced by clinicians who provide care to transgender, nonbinary, and gender diverse (TGD) young adults (aged 18–24), with a focus on unique healthcare needs and challenges.

**Methods:**

Between April 2022 – July 2022, we conducted qualitative interviews with 13 clinicians (n = 9 medical and n = 4 mental health) about perceived needs and barriers relating to the care of TGD young adults. Clinicians were recruited throughout the Southeastern and Mid-Atlantic United States and were a mix of general practitioners and specialists. Using a hybrid deductive and inductive thematic analysis approach, the interview transcripts were analyzed and key themes identified.

**Results:**

Thematic analyses of these interviews identified three main themes: the need for knowledgeable clinicians, the need and desire for reliable training resources and mentorship, and concerns surrounding the impact of the sociopolitical environment. Many participants noted a lack of access to local educational opportunities and mentorship but expressed willingness to seek these out if centralized resources, such as national platforms or accessible training modules, were available.

**Conclusion:**

This study identifies both gaps in clinician education and broader barriers – such as local politics and access to mentorship – that hinder the ability to provide effective care to TGD young adults. These findings will help to inform the development of clinician education and support programs.

## Introduction

In the United States (U.S.), an estimated 1.3% of individuals ages 18–24 years identify as transgender, and an additional percentage of U.S. young adults identify as nonbinary [1,2]. Transgender, nonbinary, and other gender diverse (TGD) young adults and their families view clinicians as a trusted source of support and information when questions arise regarding gender identity, general health, and mental health concerns [3,4]. Additionally, TGD individuals require routine preventative medical and dental services but may avoid wellness visits and other routine health maintenance if clinic practices are perceived to be unwelcoming [5].

This study focuses on TGD young adults (aged 18–24), who are in a critical developmental period characterized by social, emotional, and psychological change. This age group faces distinct challenges that differ from those experienced by adolescents or older adults – both social and legal. By concentrating on young adults, this research aims to explore the unique needs and barriers to care they encounter, which may not be fully captured in studies of broader age groups.

Access to competent medical and behavioral health care has been shown to improve mental health outcomes and psychosocial functioning for TGD young adults [6,7]. It is therefore important that clinicians understand how to have supportive interactions with TGD young adults and their families. Despite this need, many clinicians do not feel equipped to care for TGD individuals in their practices and lack the necessary educational resources and training.

While previous work has characterized some of the barriers to providing care, including a lack of formal training and limited support systems [8–9], a need persists for greater understanding of clinicians’ training gaps, goals, and desired resources to inform interventions and programming. These insights are crucial for developing targeted interventions and resources that can better equip clinicians to offer competent care. Recent studies have indicated that clinicians often feel unprepared due to limited training on care of TGD patients during their formal education [10]. These barriers are particularly evident in primary care and mental health settings. Further, clinicians’ perceived barriers to providing care may be influenced by the contemporary U.S. political climate and evolving policy environment, including the increasing number of anti-trans bills introduced that target TGD healthcare (185 bills in 2023, as compared to 40 bills in 2021 and only 4 bills in 2019) [11].

In this qualitative study, we sought to explore the past and current training experiences and needs of medical and mental health clinicians providing care to TGD young adults. This research inquiry, conducted as part of TransHealthGUIDE, a National Institutes of Health (NIH)-funded project (U01MH136558) to improve mental health outcomes for TGD young adults via innovative interventions, aimed to delineate specific gaps in the training, resources, and support systems available for clinicians caring for TGD young adults. While much of the foundational knowledge on clinicians’ needs and barriers related to providing care of TGD patients has been established [12,13], this study seeks to provide a more detailed understanding of how these challenges manifest in clinical practice, particularly for the young adult patient population. By focusing on these dynamics, the study aims to inform future interventions and program development that can more effectively equip clinicians to provide competent and compassionate care for TGD young adults.

## Methods

### Recruitment and eligibility

TransHealthGUIDE is a multi-component study that aims to improve the mental health of TGD young adults through educational interventions for health care clinicians, TGD young adults, and their family members. To identify the training experiences and needs of health care clinicians, one-on-one in-depth interviews were conducted with a total of 13 health care clinicians between April 2022 and July 2022. Eligible participants were healthcare clinicians aged 18 or older who provided care to TGD young adults, defined as individuals aged 18–24 years. Participants self-identified as providing care to this population based on their own assessment of their patient demographic, rather than a predefined threshold for practice composition. Recruitment materials specified this age range to ensure alignment with study objectives.

Purposive sampling was implemented to ensure diversity of participants by geography, specialty, and clinician type [14]. Geographic recruitment was first limited to the Southeastern U.S. because the TransHealthGUIDE study initially focused on this region. As the U.S. legislative landscape changed during the study, the geographic focus of the overall study was adjusted, and clinicians in the Mid-Atlantic were included.

Recruitment was conducted through electronic flyers, emails to organizations serving the TGD community, clinician listservs, and referrals from the research team’s national network. All study activities received approval from the Institutional Review Board at Boston Children’s Hospital (IRB-P00040048), and ethical standards were rigorously followed. Given the study’s minimal risk and the remote, geographically dispersed nature of participants, written consent was not obtained to prevent potential identification as members of a stigmatized group. Instead, the IRB approved the use of verbal informed consent, which was documented by audio-recording each participant’s verbal agreement at the beginning of the interview.

Participants received a $50 Amazon gift card as compensation for their time. Compensation was approved by the Institutional Review Board (IRB) and was not contingent on specific responses.

### Data collection

An hour-long, semi-structured, audio-recorded Zoom interview was conducted with each consenting participant. Verbal consent was obtained from all participants at the beginning of the interview. Two to four members of the study team participated as interviewers or notetakers. Participants were instructed to comment on their experiences and the care they provided to TGD young adults, defined as individuals aged 18–24. Participants were asked about their experiences caring for TGD young adults, facilitators and barriers to providing care, and resources they used to learn and teach others about caring for TGD individuals. Although some participants may have also referenced adolescents and adults due to the nature of their practices, the focus of the broader study was on young adults. Participants were also asked to self-report demographic details, including race, ethnicity, and professional background.

Audio recordings were transcribed verbatim by the study team.

### Data analysis

After transcription, data were analyzed using a hybrid deductive and inductive thematic analysis approach [15]. Initial codes were developed inductively through open coding of the first set of transcripts by two independent coders (authors MQ and EB). A preliminary codebook was iteratively refined through team discussions, incorporating deductive elements informed by prior research on TGD healthcare education. Codes were applied line-by-line, and themes were reassessed throughout the analysis.

To assess inter-rater reliability, a subset of transcripts (20%) was independently coded by MQ and EB. Coding discrepancies were reviewed in consensus meetings, and modifications to the codebook were made iteratively to improve alignment. Rather than using a statistical inter-rater reliability measure, agreement was ensured through discussion and reconciliation of coding differences, with input from additional team members (JK, RT, KK, SR, and RX) as needed. Once consistency was established, the remaining transcripts were coded by a single coder with periodic team reviews.

Themes were refined using a constant comparison approach, ensuring that newly identified patterns were consistently evaluated against prior codes across transcripts. Regular team discussions were held to compare emerging themes, refine code definitions, and resolve discrepancies. Investigator triangulation was employed, with multiple team members (MQ, EB, JK, RT, KK, SR, and RX) independently reviewing and validating key themes to ensure analytical rigor. Refinements were made iteratively based on consensus, and thematic saturation was assessed to confirm that no new patterns emerged.

The codebook included predefined themes informed by prior research, operational definitions, and example quotes. New codes emerging from the data were discussed in weekly team meetings and integrated through iterative revisions. Reliability was maintained by double-coding a subset of transcripts, with inter-coder agreement established via consensus discussions rather than statistical measures.

Qualitative coding was implemented using Dedoose software

## Results

### Sample characteristics

A total of 13 clinicians across seven states in the Mid-Atlantic and Southern U.S. were interviewed. Nine were medical clinicians, and four were mental health clinicians. Of the medical clinicians, five had MD/DO degrees (55.5%), three were nurse practitioners (33.3%), and one was a medical student (11.1%).

Six (66.7%) were general practitioners in primary care or family medicine practices, and three (33.3%) were specialists. Of the mental health clinicians, two were psychologists (50%), and two were master’s level therapists (50%). Of the 13 participants, 7 identified as White, 2 as Asian, 2 as Black, 1 as Hispanic, and 1 as multiracial.

### Themes

Themes coalesced into three broad categories: (1) the need for clinicians knowledgeable about the care of TGD young adults, (2) the need and desire for reliable learning resources to train clinicians in caring for TGD young adults and their families, and (3) concerns about the impacts of the sociopolitical environment on care delivery.

#### Theme 1: The need for knowledgeable clinicians.

This theme highlights how clinicians take initiative to educate themselves due to the absence of formal training opportunities. It encompasses two interrelated sub-themes: the need for more clinicians competent and comfortable in care for transgender people (subtheme 1.1) and the need for trusted colleagues and care networks (subtheme 1.2). In subtheme 1.1, clinicians expressed that the demand for care for transgender people exceeds the number of trained clinicians, forcing many to learn independently. In subtheme 1.2, many clinicians described seeking informal peer networks due to the absence of structured mentorship. A mental health clinician noted, “I belong to a couple of LGBTQ therapist groups on Facebook to find out who’s doing workshops, how do I sit in on this -- but it’s a lot of legwork, I would say, to collect information and collaborate (Table 1).”

**Table 1:** The need for knowledgeable clinicians.

So I first was like…I have to become trans competent….I have to make my office LGBT friendly, and I realized that it wasn’t. Not that it was unfriendly, but it just needed some more. We’re not a poster office at all. Ours is fairly formal. But we did get some LGBT heart stickers, and had a little sticker stuck in the waiting room at each of my offices, so they’re like, “hey, this is a safe place.”	Medical Clinician A
My biggest hurdle has been trying to get the insurance company [to cover care]. I mean I’ve written letters about my training and that psychologist will write letters about me. It’s just it’s a silly hurdle. It creates a barrier for care for a patient, but they don’t really care as much, you know? You try to call them [insurance companies] out, but whatever.	Medical Clinician A
I wanted to be mentored. I…wanted to be mentored really bad and I couldn’t find somebody to mentor me, and so I asked her if she would, and she agreed, and then she never followed through with allowing me to go and just see her clinic a little bit. There’s a lot to be said for just plain old mentorship.	Medical Clinician A
And, to be honest I literally thought that all you did was walk into your primary care, and say, hey I’m trans…It was completely not the case at all…	Medical Clinician B
…that experience with me calling that OBGYN’s office, and then the patient – the patient had such a horrific experience. They called our clinic crying and I talked to them on the phone for an hour to calm them down because of what this lady chose to say and do throughout her entire exam of this patient. But that’s not unusual. …I had a patient who was seeing an endocrinologist for a very complex thyroid disorder, and decided to come out to them while they were in the office. The endocrinologist literally walked them by the collar of their shirt out of their office and told them to come back whenever they had faced reality and to never dress this way in their office again.	Medical Clinician B
Now the LGBT community as a whole seeking primary care there, I haven’t heard a lot of negative [things], but for the trans community, they have a hard time accessing them. Just because of that limited [hours] being open, and that it involves a lot of volunteer work and so the provider you see this time may not be the one you see next time. And so, reaching out to them [patients], same thing, [there is] no consistency.	Medical Clinician B
It’s very hard to get word of mouth out. But I mean when it does, you know, they come immediately.	Medical Clinician B
I have a network of primary care [providers]. If I have questions or concerns, we have a network of other nurse practitioners and physicians that I can go to and inquire as far as chronic conditions. But I just don’t have that same type of resource or network when it comes to transgender [health].	Medical Clinician E
We have a huge demand. My list is probably out to maybe 3 months, and that’s only because we have this other provider who is a general [practitioner] now, but she has an interest in transgender care, so she’s going to take patients…. I try to stick patients in my schedule sort of outside of our designated gender wellness days because I know that there’s a long wait. I think our psychologist – we only have the one who does this work – I think he’s booking out into November.	Medical Clinician G
I wasn’t seeking it out. My whole philosophy is flying under the radar, and then I started to realize you can’t fly under the radar when you do this, right?	Medical Clinician I
The need has skyrocketed. I’m currently booked out at least a month, and you know I get so many calls, and it’s just a very heartbreaking thing to be like, I don’t know where to send you. I’m already squeezing in extra patients per week.	Mental Health Clinician B
…word of mouth for sure. Clients consistently come to me and say, hey, you saw my friend, or you saw my spouse, or this doctor sent me to you….The trans community in particular is so insular. It breaks my heart how many clients come to me having had incredibly negative experiences with providers. So if word gets out that, hey, there’s someone who’s pretty competent and isn’t going to treat you poorly, [clients come].	Mental Health Clinician B
…if I could have a resource base that people have been certified as being gender affirming, that would be wonderful.	Mental Health Clinician B
But mostly my training has been a lot of piecemeal and fortuitously crossing paths with providers. It’s a lot of knowledge sharing that goes on, but I seek out a lot of continuing education workshops. I belong to a couple of LGBTQ therapist groups on Facebook to find out who’s doing workshops, how do I sit in on this -- but it’s a lot of legwork, I would say, to collect information and collaborate.	Mental Health Clinician B

Subtheme 1.1: The need for more clinicians competent in caring for TGD young adults: Clinician narratives identified a significant need for more clinicians interested and competent in providing care to TGD young adults. Many clinicians discussed their own reasons for initially becoming engaged in care for transgender people. Most medical and mental health clinicians said they did not enter medical/mental health fields with plans to focus on providing care to TGD patients but were motivated to do so by a perceived unmet need for available clinicians to address the concerns of TGD young adults and their family members

Clinicians felt that this unmet need grew out of discomfort among other clinicians and because of frequent negative healthcare experiences among TGD young adults and family members seeking care in other clinical contexts. As Medical Clinician B reflected, “And, to be honest I literally thought that all you did was walk into your primary care and say, hey I’m trans… It was completely not the case at all….” This highlights the gap in understanding and the discomfort some clinicians feel when working with TGD individuals, which further contributes to the challenges in accessing competent care. Upon offering care, clinicians saw a rapid influx of TGD patients; in some cases, these patients filled their patient panels, and clinicians took on additional clinic days to meet demand for care. Clinicians explicitly noted how they created a welcoming clinic environment to indicate that the clinic was a safe place for LGBTQ+ patients. Visual safety signaling and positive interactions resulted in positive word of mouth in the community.

Subtheme 1.2: The need for trusted colleagues and care networks: Clinicians described relationships with colleagues knowledgeable about the needs of TGD young adults as essential for their own clinical practice and learning. In the absence of structured networks or formal support, many clinicians felt that it would be difficult for them to continue providing care for TGD individuals. Mental Health Clinician B described their experience: “But mostly my training has been a lot of piecemeal and fortuitously crossing paths with providers. It’s a lot of knowledge sharing that goes on, but I seek out a lot of continuing education workshops.” This highlights the challenges faced by clinicians who, without a solid network or mentorship, are left to rely on fragmented sources of knowledge and self-directed learning. These gaps in training and resources can hinder the ability to offer consistent, high-quality care to TGD individuals, underscoring the need for more coordinated support and educational frameworks.

Referral networks of trusted colleagues were important to clinicians for several reasons. First, clinicians described the value of having colleagues with whom they could discuss clinical questions related to care of TGD patients; this theme is explored further in the next section.

Second, clinicians emphasized the importance of knowing they could refer TGD patients to clinicians/practices where patients would not be stigmatized or mistreated. Some primary care clinicians further reported that insurance coverage of certain types of care would only be possible if ordered by certain subspecialists, necessitating access to subspecialists who were also competent in providing care to TGD patients.

#### Theme 2: The need and desire for reliable training resources for clinicians caring for TGD young adults and families.

This theme captures the structural challenges that prevent clinicians from accessing adequate education. It encompasses two interrelated sub-themes: challenges associated with lack of formal training programs (subtheme 2.1) and the desire for mentorship and collaboration (subtheme 2.2). Subtheme 2.1 details how medical education lacks standardized curricula for TGD healthcare, leaving gaps in clinician competency. Subtheme 2.2 details how despite the need for mentorship, many clinicians struggle to find experienced mentors. One medical clinician shared, “I wanted to be mentored. I…wanted to be mentored really bad and I couldn’t find somebody to mentor me, and so I asked her if she would, and she agreed, and then she never followed through with allowing me to go and just see her clinic a little bit. There’s a lot to be said for just plain old mentorship (Table 1).”

Subtheme 2.1: Clinician responses to the lack of formal training programs and resources: Clinicians discussed learning best practices for the care of TGD young adults by taking World Professional Association for Transgender Health (WPATH) courses; reviewing professional society guidelines, including Endocrine Society clinical practice guidelines [16], WPATH Standards of Care [17], and University of California San Francisco primary care guidelines [18]; and accessing educational resources published by the National LGBTQIA+ Health Education Center [19] (Table 2). However, many participants identified the absence of a centralized, easily accessible training platform as a barrier to developing expertise in caring for TGD young adults.

**Table 2:** The need and desire for reliable training resources for clinicians about caring for transgender, nonbinary, and gender diverse (TGD) young adults and families.

…some of the local medical schools caught on a little bit that I was doing some of this [creating handouts for patients], and so I was able to have an opportunity to do some teaching to the medical students. And when you do that, [it] really elevates your level of knowledge, because you really have to know what you’re doing.	Medical Clinician A
I mean honestly like it’s an extra step, but I feel like any provider that does my side [works as a medical provider] really needs to talk to the mental health care person. Do they even know how to diagnose gender dysphoria? Do they know what that means? And do they understand when they’re writing a letter, saying that they recommend something, do they understand what they’re recommending with the effects?	Medical Clinician A
It revealed that it wasn’t just her, but there was a lot of misunderstanding and misconception in my entire nursing staff, including my nurse manager. So I had to go and train the staff and the nurses, and I had to train everybody. I put on a couple of “lunch and learns” and then we had a little bit of a change in staff. And it’s amazing in just a couple of years how my office staff is so gung-ho…it’s just incredible like the transformation.	Medical Clinician A
Another [source of information] was a continuing education [session] that was put together by a nurse practitioner who does primary care for trans[gender] people…I contacted her, and she actually got me in touch with [resources]...[she said,] let’s do WPATH, look at the Endocrine Society. She was a really good guide at first.	Medical Clinician B
Clinical contacts, that’s where I learned most of the information. Because that’s when you really get to sit down with a patient, create a treatment plan, and see them come back for a follow-up	Medical Clinician C
All of those [information sheets and flyers] – we wanted a PrEP flyer, so we gathered information from the CDC and created our own PrEP flyer.	Medical Clinician D
[I find information through]…literature, looking at the evidence out there now. Also, if I knew other practitioners who work with [the] transgender population, that [would] be a great resource.	Medical Clinician E
So I didn’t really get much exposure during residency at the gender clinic because I think it was just getting started up, but definitely during fellowship. We rotated through the gender clinic, we had lectures, went to meetings and did all of that. A lot of normal fellow apprenticeship type stuff where you like work with [the founding doctor].	Medical Clinician F
So, I am practicing as a [specialist] there but…I don’t have psychology or social work, and because of insurance issues, they can’t come see me at [my institution]. So luckily, some of the hospitals in [State] are starting to expand their programs…, but I made it a point to not see those patients in [State] because I don’t have the support that I need there and the patients don’t have the support they need there.	Medical Clinician G
Back then, circa 2013, they didn’t even have any online training modules or anything, but I read WPATH cover to cover, the Endo[crine] Society guidelines at that time. More recently, the UCSF guidelines [are what] I find the most helpful, and I think I came across that in 2014 or 2015, maybe. But that’s my go to resource these days. That’s where I would start if I had a question.	Medical Clinician H
I continued to educate myself, read all the guidelines. I talked with some experts.	Medical Clinician H
Pretty much self-trained in residency. I created an elective for myself and went to the [Health Center] for an elective, learned a lot there.	Medical Clinician H
I just called people when I had questions, and now I’m an expert, and I trained my 3 nurse practitioners. One of them had done some gender-affirming care, and the other two hadn’t done any. And so I personally trained all of them to do it the way that I think it should be done.	Medical Clinician H
WPATH, for example – their training thing...I’ve never actually done because I read what was involved, and I was like, I don’t have time for that. I really don’t.	Medical Clinician H
I think what I’ve learned because I’m very much very clinical is that, clinical experience, even if it’s not published, is very valuable, and that’s just across everything I do.	Medical Clinician I
I got it through my fellowship at [a different institution]. I rotated with [two doctors] and did all that through them. I think, you know, as I became an attending, I did attend some WPATH sessions and GEI…I’m not fully certified yet but I’ve started that process. And continuing my medical education after my fellowship.	Medical Clinician I
And you know, sometimes I rely on families for resources, because I have one mom who gave me a whole list of books that she read that helped her and another family was part of forming like a support group in a rural area.	Medical Clinician I
Not only do I have to get my 20 hours a year to maintain licensure, but I’m also collecting lots of hours that aren’t accredited, and so I see some push back. I don’t fault providers because our time is really limited. So if they’re like, “Okay, I need to get 20 hours of training, I would like to do LGBTQ stuff. But if one is accredited and I get licensure hours for it, well, I’m going to do that.” I would say it’s probably only about 50/50 of the time that some of the continuing ed[ucation] and workshops I do are accredited.	Mental Health Clinician B
So I come across lots of providers who [have] really good hearts, and really want to offer this care, but either don’t have or don’t know how to get the education. I run into that a lot with clients, who are like, I can’t be the one to teach my therapist what I need or how to do this.	Mental Health Clinician B
Absolutely, [cost of CMEs is a real big barrier] because the training I did was $2,500, which is a lot. And I got so excited about it. I was kind of being a cheerleader for them [the training organization] and trying to get other counselors to do this [whom] I knew were allies – and as soon as they heard the cost they said, no, I can’t afford it. So cost is a huge barrier.	Mental Health Clinician C
I think just how to kind of come more so from like a systems perspective, with getting the family on board, with helping the family be an advocate…, and then helping them find those medical resources.	Mental Health Clinician C
I could tell I wasn’t being the best counselor in that moment, because a lot of kind of the questions [I had] would be part of my curiosity or my education. Which we all know, our clients are not supposed to teach us things, we’re supposed to teach them. So, that’s when I kind of said, let me put a pause on this and find out more.	Mental Health Clinician C

Several described developing or feeling motivated to create self-guided training programs and/or their own educational resources, drawing from existing guidelines and multiple professional societies. Clinicians also emphasized the importance of clinical experiences, both formal and informal, with colleagues and training programs. Further, some noted that the lack of professional continuing education credits was a deterrent to accessing certain educational resources.

Subtheme 2.2: The desire for mentorship/collaboration: While clinicians expressed strong interest in mentorship and learning from colleagues, some were unable to establish these relationships. Those who were able to do so reported positive experiences related to receiving clinical instruction from more knowledgeable peers and mentors and in turn mentoring others. Those who went on to teach peers and trainees were particularly emphatic about the importance of developing a strong understanding of the material.

Professional training courses were identified as useful opportunities to learn from colleagues with experience providing care to TGD young adults, but cost and required time commitment were noted as barriers to access. Several clinicians participated in the care of TGD young adults during their training, but others reported that colleagues interested in providing care to TGD individuals have been hindered by a paucity of training opportunities.

Subtheme 2.3 The need for multidisciplinary teams: Medical and mental health clinicians were enthusiastic about cross-disciplinary educational opportunities encompassing medical specialists, mental health clinicians, and administrative staff. Clinicians wanted to ensure that patients had access to specialty and mental health care beyond their own practices. They also emphasized supporting families to access resources and care.

Clinicians expressed desire for more opportunities to educate clinic staff, including nurses, clinical assistants, and front desk staff. Some clinicians also noted that encounters with TGD young adults and families in their practice helped them to identify educational resources and highlighted gaps in existing knowledge.

#### Theme 3: Sociopolitical environment.

Clinicians identified the U.S. legislative environment as a barrier to providing care for TGD individuals, citing uncertainty about legal implications. Proposal or passage of legislation relating to care for transgender people was a concern for multiple clinicians, who reported needing to understand frequent changes in the status of legislation (Table 3). Clinicians in states without legislation relating to care for transgender people also expressed concerns about managing patient demand. Mental Health Clinician B explained, “We’re already so busy with people in our own state, how are we going to handle it if we’re getting an influx from other states? How do we keep up with the demand? I’m hearing nervousness and concern kind of on both sides.” This sentiment underscores the strain on healthcare systems and the growing anxiety among clinicians who may face an increase in patient volumes from states with less supportive environments or fewer legal protections for TGD individuals.

**Table 3:** Sociopolitical environment.

I’ve worried, because things are kind of heating up here in [State]. So I’ve got my eyes open. Let’s just say that.	Medical Clinician A
We get calls almost weekly – on a weekly basis, and especially with [state] closing down... We have- we’ve contacted lawyers. [Advocacy organization] …. They haven’t gotten us back because they just don’t know what to do, because it’s like – I Imagine it’s an unprecedented case, you know?	Medical Clinician B
No reason not to do it [provide care] except for you’re either scared – which is understandable, like I said, we do get death threats fairly regularly – and you could be scared for your career.	Medical Clinician B
We have contacted lawyers, [advocacy organization], and then personal lawyers. They haven’t gotten us back because they just don’t know what to do, because it’s an unprecedented case. What would be our legal liability with [state]? I don’t know. So hopefully we’ll get something on that soon.	Medical Clinician B
I’m not going to change what I’m doing [providing care] unless [my institution] makes us. I know we’re not changing anything right now, but we have heard of colleagues – a couple of private practice type people – who have stopped taking new patients… it’s not going to make me not practice in this area.	Medical Clinician F
My approach is I don’t watch the news. I haven’t read, watched, listened to the news for over a year, it’s been great…. Because I mean it’s for my own mental health. I probably wouldn’t do my job if I paid attention, so I just don’t.	Medical Clinician H
I feel like the people who are doing it [providing care] are people who have had some training in it, and then everybody else is like, I’m not doing this, just because of the environment we’re in.	Medical Clinician I
[State-based medical organization] reached out to me about the need for testimony at the State House… It would be helpful if there were [resources for] people who aren’t even members of [national medical organization] to have something available or someone to talk to about it [legislation] because it‘s terrifying, you know?	Medical Clinician I
We’re already so busy with people in our own state, how are we going to handle it if we’re getting an influx from other states? How do we keep up with the demand? I’m hearing nervousness and concern kind of on both sides.	Mental Health Clinician B

While the study focuses on the needs of young adults (aged 18–24), some clinicians referenced concerns about their broader clinical practices, spanning a wider age range.

Multiple clinicians discussed the fear that threats related to providing care to TGD individuals represented a significant barrier for other clinicians who might otherwise wish to develop further expertise. Clinicians also expressed concern about the potential strain on resources resulting from an influx of patients and the lack of clarity about whether providing care to out-of-state patients would be legal. Despite the uncertainty of the legislative environment, some clinicians expressed an interest in continuing to provide care for TGD patients.

Several clinicians discussed their involvement in state-based advocacy and national professional organizations. Additionally, clinicians expressed interest in receiving training regarding advocacy and understanding the potential impacts of legislation on their practice.

## Discussion

This qualitative study employed a semi-structured interview design to explore the educational landscape for medical and mental health clinicians caring for TGD young adults. A purposive sampling strategy was used to recruit participants with varying levels of experience in care for transgender people, ensuring a diverse range of insights. Thematic analysis of clinician interviews identified key barriers to education for clinicians interested in caring for TGD individuals, including lack of formal training in traditional educational settings, limited access to mentorship, and inconsistent institutional support. This study advances understanding of many of the barriers to care for TGD young adults and their families.

A major challenge facing clinicians who care for TGD individuals is the lack of formal training in medical and mental health education programs. Research indicates that most medical schools allocate fewer than five hours to LGBTQ+ health content, often without specific coverage of transgender care [20,21]. While previous research has identified similar barriers [8,9,22], this study is unique in demonstrating that clinicians are willing to seek out and undergo systematic training outside of their local practice environment to address gaps in their formal medical education regarding care for transgender people. Many clinicians interested in developing expertise in the care for TGD populations have committed considerable time to developing self-directed learning plans, synthesizing guidelines across multiple professional societies, and identifying their own professional networks for training and patient referral. This initiative is often motivated by patient needs, personal commitment to inclusivity, and frustration with inadequate institutional training. However, self-directed learning presents challenges, including limited access to high-quality resources, time constraints, and a lack of structured mentorship.

Beyond initial training, professional development opportunities remain limited. Many CME-programs focusing on transgender healthcare are either cost-prohibitive or inaccessible, particularly in states with restrictive policies. Even when training opportunities exist, clinicians report a lack of accreditation or formal institutional support, making it difficult to justify the time investment. Additionally, cultural competence training is often insufficient, with many clinicians lacking exposure to TGD patients during their clinical education. Without structured learning opportunities, clinicians may struggle with inclusive communication, appropriate medical decision-making, and understanding the broader psychosocial needs of TGD individuals.

The study findings underscore the importance of including information on caring for TGD people in undergraduate and graduate medical and health professions education [23] and highlight an opportunity to develop accessible, high-quality, and centralized training programs, resources, and mentorship opportunities. Building on our findings, we recommend that TGD healthcare training programs incorporate standardized, competency-based curricula into medical and mental health education. Structured training should include case-based learning, simulated patient interactions, and interdisciplinary collaboration. Additionally, expanding accessible CME opportunities and fostering the development of clinician mentorship networks will improve clinician competency.

Importantly, participants in this study expressed a strong desire for cross-disciplinary educational opportunities and for fostering multidisciplinary networks. Connecting education across fields – such as nursing, social work, psychology, and medicine – may enhance continuity and coordination of care, ultimately improving healthcare experiences for TGD individuals. Interdisciplinary collaboration not only strengthens clinician competency but also fosters comprehensive care models that integrate medical, mental health, and social support services, ensuring a more holistic approach to care that is consistent with TGD patients’ self-articulated healthcare needs [24]. By reducing the burden on individual clinicians, these strategies may enhance clinician confidence, improve patient care, and ensure equitable access for TGD populations.

Moreover, an intersectional lens is useful for analyzing the ways race, ethnicity, class, religion, and immigration status inform TGD healthcare experiences. Structural inequities in healthcare may be reinforced through curriculum bias and inadequate cultural competency training, which fail to address the barriers faced by TGD individuals from marginalized communities. These systemic shortcomings could exacerbate healthcare disparities if clinicians are unprepared to deliver culturally responsive and equitable care. Addressing these issues requires updating curricula to include intersectional perspectives and implementing practices that promote equitable care for all TGD individuals.

The present study also provides valuable insight into the specific impacts of U.S. legislation on clinician education. Concerns about the impact of legislation on availability of compassionate and competent care for TGD populations are consistent with previous published findings [25]. Findings from this study suggest that the legislative landscape significantly impacts the ability of clinicians to provide care for TGD individuals. Recent laws in several Southern and Mid-Atlantic states have restricted access to certain forms of care for these populations. Even in states where care remains legal, clinicians still face challenges, including insurance denials and administrative burdens that limit service availability.

Beyond direct care restrictions, legal uncertainties have created a chilling effect among healthcare professionals. The risk of criminal or licensure consequences, lack of clarity or understanding about the nuances of the legal landscape in each state, and the possibility of new legislation being proposed or passed have led many clinicians to withdraw from care of TGD populations, exacerbating workforce shortages. Despite increasing demand for clinicians knowledgeable about the general health and mental health needs of TGD young adults, clinicians who would otherwise be interested in learning about care for TGD individuals may be deterred by active or pending legislation. Institutions may also hesitate to offer TGD healthcare training due to political pressures, further restricting clinician competency development. Addressing these barriers will be critical to ensuring equitable access to care and supporting clinicians committed to serving TGD populations.

A growing need exists for clinicians who can care for TGD young adults and their families. Training clinicians to engage effectively with and identify the needs of TGD individuals is essential to address the increased risk of mental health problems in this population. Bridging this gap will require efforts on multiple fronts. First, additional rigorous research is needed to define best practices for the care of TGD young adults. Second, relevant clinical care competencies must be delineated for clinicians who see TGD young adults in their practice. For example, managing patients with questions and concerns about gender identity may require skills for engaging in conversations about gender, creating a welcoming clinic environment, establishing a relationship of trust so that patients are more likely to comply with routine health maintenance, and facilitating referrals to other trusted clinicians when appropriate. Third, evidence-based and accessible training resources need to be developed to support clinicians in achieving these clinical competencies. Fourth, facilitating mentorship relationships among interested clinicians can further support professional development.

This study uniquely highlights the perspectives of a small but diverse group of medical and mental health clinicians with variable levels of training. The study contributes to the existing literature by emphasizing the willingness of clinicians to engage in self-directed learning and the challenges they face in doing so. Unlike previous research that primarily documents systemic barriers [26,27], this study offers insights into the personal motivations, resourcefulness, and adaptability of clinicians who seek to bridge their own educational gaps. These findings underscore the need for scalable, accessible, and competency-based training solutions tailored to the realities of clinicians working in diverse clinical settings.

Addressing these barriers requires systemic efforts to educate and support clinicians to provide compassionate and competent care to TGD populations. Expanding structured, accredited medical education on TGD healthcare may help counteract gaps in clinician preparedness. As policy landscapes continue to evolve, strategies are also needed to ensure that healthcare professionals are supported to understand the implications of legislation and tailor their care appropriately.

### Limitations

This study has several limitations. The sample size is small but appropriate for the goals of this qualitative inquiry [28]. While financial incentives may influence participation, the compensation for this study was modest, and efforts were made to recruit a diverse sample of clinicians with varying levels of experience in care for transgender people. Given the purposive sampling approach, this is a non-representative sample by design. Clinicians who participated in the study likely have more interest in, exposure to, and experience with managing TGD patients in their practice as compared to many general practitioners. As such, their perspectives may not be generalizable.

This study included a variety of clinician types across different stages of training and experience (e.g., fellowship-trained clinicians, primary care clinicians, mental health professionals, and one medical student) to gauge a variety of perspectives on educational challenges. The experiences and educational needs of these diverse clinician groups nevertheless may not be representative of the overall healthcare clinician population, as their training, responsibilities, and exposure to care for transgender people vary widely. For example, fellowship-trained clinicians have extensive experience but typically focus on a single specialty, while general practitioners may address a more diverse array of medical and mental health concerns. The medical student was in the early stages of training, lending a different perspective on care needs and barriers.

Additionally, the relatively small number of participants may have affected thematic and theoretical saturation. While some degree of saturation was reached, the diversity in clinician types makes it difficult to fully determine whether all relevant themes were explored. Larger studies with more homogenous groups of clinicians, or stratified analyses based on clinician experience and training, could help to validate and expand the understanding of educational needs and barriers.

Gender identity data were not systematically collected from participants. Future studies should consider including gender identity data to better understand how clinician gender identity influences experiences in TGD healthcare.

Nevertheless, the findings of this study highlight many of the concerns and barriers to training that other clinicians are likely to experience. General practitioners who encounter TGD young adults in their practices must be equipped with the knowledge and skills to conduct informed conversations, assess and triage health needs, and direct patients and families to appropriate resources. This study underscores the importance of understanding clinicians’ unmet educational needs and developing effective and accessible clinician training tools to address them.

## Conclusion

There is a growing need for clinicians knowledgeable about the mental health needs of TGD young adults, yet gaps exist in clinician education and training. Barriers to addressing these gaps include lack of reliable and accessible training sources, lack of mentorship, and a changing sociopolitical environment. Many clinicians are interested in and willing to seek out training and mentorship opportunities. These findings will help to inform the development of clinician training curricula at a national level.
